# Warping of Rubber

**Published:** 1904-11

**Authors:** 


Warping of Rubber.—When plaster of Paris is used for investment, warping of vulcanite in cooling is very apt to occur if rapid cooling is effected. The writer had a case where the plate split in the vulcanizer with a noise that could be distinctly heard. This was a "hurry job," the investment being used while quite new, and water was being poured on the vulcanizer to hasten cooling at the time the break occurred. The crack commenced at the posterior border and extended about an inch along the palate; one of the two wings thus made being so warped as to be almost at a rectangle with the other. This unmistakably demonstrated to me the liability of plates to warp from rapid cooling with a plaster-of-Paris investment. And yet a moderate amount of warpage might possibly produce a good fit. It is all a game of chance if Paris plaster is used for impression, model and investment.—*Brief.*

